# Peripheral vascular response to inspiratory breath hold in paediatric homozygous sickle cell disease

**DOI:** 10.1113/expphysiol.2011.064055

**Published:** 2012-06-01

**Authors:** Veline S L’Esperance, Sharon E Cox, David Simpson, Carolyn Gill, Julie Makani, Deogratias Soka, Josephine Mgaya, Fenella J Kirkham, Geraldine F Clough

**Affiliations:** 1 Vascular Research Group, Human Development and HealthSouthampton, UK; 5 Clinical Neurosciences Division, Faculty of Medicine, University of SouthamptonSouthampton, UK; 2 MRC International Nutrition Group, London School of Hygiene & Tropical MedicineLondon, UK; 3 Institute of Sound and Vibration Research, Faculty of Engineering and the Environment, University of SouthamptonSouthampton, UK; 4 Muhimbili University of Health and Allied SciencesDar-es-Salaam, Tanzania; 6 Neurosciences Unit, UCL Institute of Child HealthLondon, UK

## Abstract

**New Findings:**

**• What is the central question of this study?:**

Autonomic nervous dysfunction is implicated in complications of sickle cell anaemia (SCA). In healthy adults, a deep inspiratory breath hold (IBH) elicits rapid transient SNS- mediated vasoconstriction detectable using Laser Doppler Flux (LDF) assessment of the finger-tip cutaneous micovasculature.

**• What is the main finding and its importance?:**

We demonstrate significantly increased resting peripheral blood flow and sympathetic activity in African children with SCA compared to sibling controls and increased sympathetic stimulation in response to vasoprovocation with DIG.

This study is the first to observe an inverse association between resting peripheral blood flow and haemoglobin oxygen saturation (SpO2). These phenomena may be an adaptive response to the hypoxic exposure in SCA.

There is increasing evidence that autonomic dysfunction in adults with homozygous sickle cell (haemoglobin SS) disease is associated with enhanced autonomic nervous system-mediated control of microvascular perfusion. However, it is unclear whether such differences are detectable in children with SS disease. We studied 65 children with SS disease [38 boys; median age 7.2 (interquartile range 5.1–10.6) years] and 20 control children without symptoms of SS disease [8 boys; 8.7 (5.5–10.8) years] and recorded mean arterial blood pressure (ABP) and daytime haemoglobin oxygen saturation (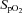). Cutaneous blood flux at rest (RBF) and during the sympathetically activated vasoconstrictor response to inspiratory breath hold (IBH) were measured in the finger pulp of the non-dominant hand using laser Doppler fluximetry. Local factors mediating flow motion were assessed by power spectral density analysis of the oscillatory components of the laser Doppler signal. The RBF measured across the two study groups was negatively associated with age (*r*=−0.25, *P* < 0.0001), ABP (*r*=−0.27, *P*= 0.02) and daytime 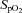 (*r*=−0.30, *P*= 0.005). Children with SS disease had a higher RBF (*P*= 0.005) and enhanced vasoconstrictor response to IBH (*P*= 0.002) compared with control children. In children with SS disease, higher RBF was associated with an increase in the sympathetic interval (*r*=−0.28, *P*= 0.022). The SS disease status, daytime 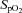 and age explained 22% of the variance in vasoconstrictor response to IBH (*P* < 0.0001). Our findings suggest that blood flow and blood flow responses in the skin of young African children with SS disease differ from those of healthy control children, with increased resting peripheral blood flow and increased sympathetic stimulation from a young age in SS disease. They further suggest that the laser Doppler flowmetry technique with inspiratory breath hold manoeuvre appears to be robust for use in young children with SS disease, to explore interactions between 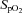, ABP and autonomic function with clinical complications, e.g. skin ulceration.

Haemoglobin SS disease (SS disease) is the most common monogenetic condition in the world and is due to homozygosity for a point mutation in the gene encoding the β-globin chain of haemoglobin, causing the amino acid glutamic acid to be replaced with valine at the sixth position. In SS disease, normal adult haemoglobin is replaced with sickle haemoglobin, which polymerizes when deoxygenated, distorting the red cells into a sickle shape that can occlude the microvasculature. The aetiology of the wide range of acute and chronic complications of SS disease remains unexplained, but there is an association with the degree of haemoglobin oxygen desaturation (Quinn *et al.* 2009), and alterations in the peripheral vasculature may be involved (Mohan *et al.* 1998,2011; Sangkatumvong *et al.* 2011).

Exposure to cold, fever and dehydration increase the risk of painful vaso-occlusive episodes, a common complication in SS disease, possibly because of increased sympathetic drive in response to these environmental triggers causing vasoconstriction. The consequent decrease in local perfusion and increase in transit time mean that the sickle cells with deoxygenated haemoglobin remain longer in the microcirculation, where the haemoglobin is more likely to polymerize and the cells to sickle (Sangkatumvong *et al.* 2011). Exaggerated sympathetic nervous system responses have been documented in children with SS disease, particularly those with severe disease, i.e. with more complications (Pearson *et al.* 2005), and the involvement of autonomic nervous dysfunction has been implicated in sudden death in SS disease (Romero *et al.* 1997; Connes *et al*. 2006). Autonomic dysfunction has also been demonstrated in adults with SS disease, and several research groups have suggested that painful crises may reflect exaggerated sympathetically evoked responses in bone marrow, evoked by cooling and other environmental stimuli (Mohan *et al.* 1998). Furthermore, autonomic dysfunction is more common in adults with SS disease with skin ulcers (Mohan *et al.* 1997), suggesting a potentially modifiable pathophysiological mechanism.

Recently, the cutaneous circulation has emerged as an accessible and potentially representative vascular bed in which to examine the mechanisms that control microvascular function. It has been argued that pathology-induced changes in cutaneous microvascular function may reflect those occurring in less accessible vascular beds and thus provide a useful surrogate in which to investigate deficits in microvascular function (Holowatz *et al.* 2008), including those found in obesity-associated insulin resistance (Clough *et al.* 2011; De Boer *et al.* 2012), diabetes (Clough *et al.* 2011), hypertension, (Gryglewska *et al.* 2010; Rossi *et al.* 2011) and ageing (Gooding *et al.* 2006; Avery *et al.* 2009) and in smokers (Avery *et al.* 2009).

Laser Doppler flowmetry is a widely used non-invasive method used to assess dynamic changes in blood flux in response to vasoreactive stimuli in both health and disease (Roustit & Cracowski, 2012). While laser Doppler flowmetry has been used previously to assess peripheral vascular autonomic dysfunction (Young *et al.* 2006; Quattrini *et al.* 2007) and vasoconstrictive episodes (Rauh *et al*. 2003), it has seen limited use to date in the measurement of perturbations in blood flux in individuals with SS disease (Sangkatumvong et al. 2011; Mohan *et al*. 2011). Digital power spectral analysis of frequency domains of the laser Doppler signal allows investigation of the rhythmic variations in skin perfusion (flow motion) related to endothelial activity (Stefanovska *et al.* 1999), myogenic activity of vascular smooth muscle (Colantuoni *et al.* 1994) and local sympathetic activity (Söderström *et al*. 2003), as well as higher frequency haemodynamic and respiratory influences (Bollinger *et al*. 1993; Rossi *et al.* 2011; Sheppard *et al.* 2011). Previous studies using spectral analysis of spontaneous microvascular fluctuations have demonstrated reduced activity in the ∼0.1 Hz (sympathetic) frequency band in individuals with insulin-dependent diabetes and impaired autonomic function (Bernardi *et al*. 1997) and have been shown to correlate with disturbances in cardiac autonomic tests, which are at least in part under sympathetic control (Meyer *et al*. 2003). Application of such approaches in SS disease has yet to be explored.

In healthy adults, an inspiratory breath hold (IBH) can elicit a rapid and transient sympathetically mediated vasoconstriction that can be detected in the cutaneous micovasculature of the finger tip pulp (Binet & Sollier, 1895; Allen *et al.* 2002; Rauh *et al.* 2003; Feger & Braune, 2005). Previous studies have used the vasoconstrictor response to IBH to investigate peripheral sympathetic deficits in autonomic failure (Young *et al.* 2006), diabetic neuropathy (Quattrini *et al.* 2007) and Fabry disease (Møller *et al*. 2009). Although well validated, the inspiratory breath hold stimulus with laser Doppler flowmetry to evoke a skin vasomotor response has rarely been explored in children or adults with SS disease, and there are few data on any association with haemoglobin oxygen desaturation (Sangkatumvong *et al*. 2011).

The aim of this study was to evaluate the skin vascular response to inspiratory breath hold in African children with SS disease compared with age-matched control children, to determine whether there are differences in sympathetically mediated control of skin blood flow between these two groups and to investigate associations between sympathetically mediated control of skin blood flow and other SS disease-associated pathophysiological parameters.

## Methods

Children with SS disease who were routinely seen at the Sickle Cell Disease Clinic at the Muhimbili National Hospital, Dar-es-Salaam, Tanzania, and sibling controls were recruited. The SS disease had been confirmed by haemoglobin electrophoresis in the children with symptomatic SS disease, but we did not have ethical permission to obtain blood samples from the asymptomatic control children to exclude SS disease. The children assented and their parents gave fully informed written consent in Kiswahili to participate in the study, which was approved by the Muhimbili University of Health and Allied Sciences committee (reference MU/01/1022/081/19). Patients were confirmed to have SS disease by HPLC of blood samples and met the following criteria for steady state: temperature ≤37.4°C; negative malaria rapid test; absence of pain; and no hospital admission or blood transfusion within 90 days. Body mass index (BMI) z-scores and centiles for sex and age were derived against the US CDC 2000 reference (Kuczmarski *et al*. 2000). Mean arterial blood pressure (ABP) was measured with a cuff placed on the non-dominant arm at the level of the heart (Dinamap Pro 400 V2 Monitor, GE Medical Systems Information Technologies, Inc., Milwaukee, WI, USA), and haemoglobin oxygen saturation (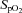) was measured using motion-resistant pulse oximetry during the day at rest using a 2 s averaging time and 1 Hz sampling rate (Masimo Radical, Irvine, CA, USA; Artemis Medical, London, UK).

**Figure 1:**
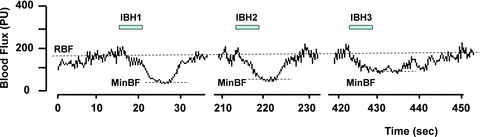
Skin blood flux in the pulp of the index finger of the non-dominant hand measured using laser Doppler fluximetry before and during three inspiratory breath holds (IBHs) each lasting 6 s  The start of each IBH is marked by the bar. A period of 3 min was allowed between each IBH. Abbreviations: RBF, resting blood flux; and MinBF, minimal blood flux.

Skin blood flux was measured at rest and during IBH by laser Doppler fluximetry (moorVMS-LDF; Moor Instruments UK, Axminster, UK) using a Class 1, 2.5 mW 785 nm wavelength laser and 0.5 mm fibre separation combined optical and temperature probe (VP1T; Moor Instruments UK). Studies were conducted in a quiet, temperature-controlled room (24 ± 1°C), with the subject in a semi-recumbent position and the arm supported at heart level. The probe was mounted on the skin of the pulp of the index finger of the non-dominant hand. Following acclimation to room surroundings, blood flux was recorded continuously for up to 15 min before and during three 6 s duration IBHs (Fig. 1). In order to obtain a reproducible vasoconstrictor response to inspiratory breath hold, the children were instructed to breathe in rapidly through their nose and to hold their breath for 6 s as counted by the investigator. The children were then instructed to release the breath hold and breathe out gently. This was repeated three times, with a period of 3 min between each IBH. Each child was allowed to practise the IBH manoeuvre during the period of acclimation and before positioning of the flux probe. Red cell flux (RCF) was measured as the mean blood flux over 5 min prior to the IBH manoeuvre, calculated using the manufacturer's software (Moor Instruments UK) and expressed in perfusion units (PU). The minimal blood flux in response to the IBH was taken as the minimal blood flux measured over 5 s of trace following the manoeuvre (Fig. 1). Data were rejected where the blood flux trace precluded identification of this minimum; usually as a result of a movement artefact during termination of the IBH. As it was noted that in some individuals the blood flux following each IBH did not return to RCF during the 3 min interval between IBHs, the fall in blood flux in response to IBH was calculated as the difference between the minimal blood flux and that measured immediately prior to each IBH (Fig. 1). A mean value for the fall in blood flux in response to IBH was estimated and the vasoconstrictor response for each participant presented as the mean IBH response expressed as a percentage of resting blood flux. The coefficient of variation in the repeated IBH response in 20 healthy children was 5.9%.

In order to explore the frequency components of the laser Doppler flowmetry signal attributed to local flow motion within the vascular bed, the continuously recorded resting laser Doppler flowmetry signal data (minimum of 10 min) was captured at a sampling rate of 40 Hz and processed as described previously using a fast Fourier transform (Clough *et al.* 2009). The frequency bands investigated in this study were those reflecting control of flow motion through myogenic activity (III, 0.05–0.15 Hz), local sympathetic activity (IV, 0.02–0.05 Hz) and endothelial activity (V, 0.0085–0.02 Hz; and VII, 0.005–0.0085 Hz; Stefanovska *et al.* 1999). The absolute value of power spectral density (PSD) within each band was expressed relative to total spectral power summed across the frequency band 0.005–0.15 Hz (Clough *et al.* 2009).

### Statistical analysis

Statistical analyses were performed using SPSS for Windows version 17.0 (SPSS Inc., Chicago IL, USA). All the data were normalized and are presented as medians and interquartile ranges (IQRs) or 95% confidence intervals (CIs). To compare groups, the Mann–Whitney *U* test was used. Regression coefficients are presented for univariate linear regression analyses of normally distributed data. Multivariable linear regression models were developed to describe factors that were independently associated, with vasoconstrictor response to IBH as the dependent (outcome) variable. Factors that were entered into the multivariable linear regression model as explanatory variables were chosen from the results of univariate analysis and included age, BMI z-score, mean and systolic ABP and daytime 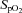. To test whether there was an independent effect of sickle cell status and sex in the model, sex and SS disease status were also included in these models as binary indicator variables. A *P* value of <0.05 was considered statistically significant for all analyses.

## Results

The characteristics of the study groups are summarized in Table 1. Sixty-five children with SS disease (38 boys) of median age 7.2 years (IQR 5.1–10.6 years) and 20 control children (8 boys) with no symptoms of SS disease and a median age of 8.7 years (IQR 5.5–10.8 years) were studied. The SS disease and control study groups did not differ with respect to age, sex or BMI z-score, but there were significant between group differences in mean arterial blood pressure, systolic blood pressure and daytime 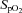 (*P* < 0.05). Children with SS disease had a median haemoglobin of 7.1 g dl^−1^ (IQR 6.65–7.8 g dl^−1^) and mean cell volume of 81 fL (IQR 75–87); haematology data were not available from the control group.

**Table 1:** Basic characteristics of study groups

Variables (medians and interquartile ranges)	Homozygous SS [*n*= 65; 38 (58%) male]	Asymptomatic controls [*n*= 20; 8 (40%) male]	Group difference (*P* value)
Age (years)	7.7 (2.7–12.5)	8.7 (5.5–10.8)	0.634
Body mass index z-score	−0.93 (−1.68–0.875)	−0.31 (−1.48–0.08)	0.154
Haemoglobin oxygen saturation (%)	97 (94.0–99.8)	100 (98.0–100)	0.001
Mean arterial blood pressure (mmHg)	71 (64.0–77.3)	79.5 (72.1–83)	0.005
Resting blood flux (PU)	303 (255–338)	234 (188–294)	0.005
Skin temperature (°C)	35.5 (35.0–36.4)	35.6 (34.2–36.2)	0.41

Results are expressed as means (95% confidence intervals).

**Figure 2:**
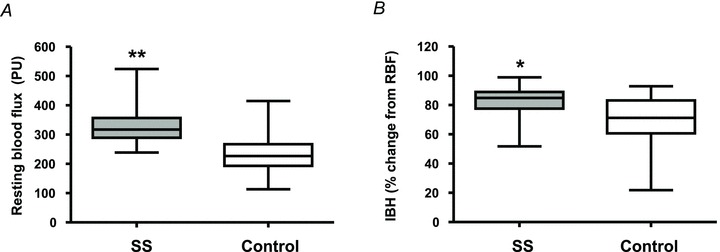
Mean resting blood flux (RBF; *A*) and mean change in blood flux during inspiratory breath hold (IBH; *B*) expressed as a percentage change from baseline measured in African children with sickle cell (SS disease) (*n*= 65) and control children (*n*= 20)  Data are expressed as the median, range and 95% confidence interval; *P= 0.002 and **P < 0.0001.

Resting laser Doppler blood flux (RBF) was significantly higher in children with SS disease compared with control children (*P*= 0.005; Table 1 and Fig. 2). In univariate linear regression analysis, RBF measured across the two study groups was negatively associated with age (*r*=−0.25, *P* < 0.0001), mean ABP [*r*=−0.27, *P*= 0.02] and daytime 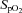 (*r*=−0.30, *P*= 0.005; see Fig. S1 (http://onlinelibrary.wiley.com/doi/10.1113/expphysiol.2011.064055/suppinfo) for scatter plots of the data). There was no significant difference in total power (0.005–0.15 Hz) between the two study groups or in the relative PSD of the four spectral power bands reflecting local flow motion control (III–VI; Table 2). In children with SS disease, RBF was positively associated with the relative spectral power in the sympathetic PSD band (*r*=−0.28, *P*= 0.0022) and negatively with the myogenic frequency band [*r*=−0.30, *P*= 0.018; Fig. S2 (http://onlinelibrary.wiley.com/doi/10.1113/expphysiol.2011.064055/suppinfo)]. There was no significant correlation between RBF and PSD in those bands in control participants. Neither RBF nor PSD in the sympathetic band was related to haemoglobin in the SS disease cohort.

**Table 2:** Comparison of total spectral power (0.005–0.15 Hz) and relative spectral power (with regard to total power) of the resting skin blood flow motion frequency bands in children with sickle cell (SS) disease compared with control children

Spectral power band	SS disease	Control
VI (0.005–0.0085 Hz)	0.14 (0.13–0.17)	0.12 (0.10–0.20)
V (0.0085–0.02 Hz)	0.24 (0.23–0.28)	0.24 (0.20–0.30)
IV (0.02–0.05 Hz)	0.27 (0.26–0.30)	0.28 (0.25–0.32)
III (0.05–0.15 Hz)	0.30 (0.28–0.35)	0.28 (0.25–0.36)
Total power (0.005–0.15 Hz)	213 (106–393)	312 (69– 792)

Results are expressed as medians (95% confidence interval) for *n*= 65 SS disease and *n*= 20 control children. No significant differences were observed in any frequency SS disease *versus* control.

The IBH manoeuvre was successfully undertaken by all but one participant aged 2.8 years, who was unable to undertake the breath hold. Additionally, in 12 participants one of the three IBH responses was excluded due to movement artefacts during the manoeuvre or an unsuccessful breath hold. Inspiratory breath hold elicited a distinct vasoconstrictor response in the skin of the finger pulp in SS disease and control participants. The minimal blood flux achieved during IBH was significantly lower in children with SS disease [47 (32–66) PU] compared with control children [62 (45–106) PU; *P*= 0.003]. The vasoconstrictor response to IBH (calculated as mean fall in blood flux during IBH expressed as as a percentage of resting blood flux) was enhanced in children with SS disease [85 (76–89)%] compared with control children [71 (61–83)%; *P*= 0.002; Fig. 2). In univariable linear regression the vasoconstrictor response to IBH was negatively associated with daytime 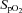 (*r*=−0.31, *P*= 0.003). In a multivariable linear regression model including SS disease status, daytime 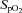 and age, the vasoconstrictor response to IBH was independently associated with SS disease status (β coefficient =−0.41, *P* < 0.0001) and there was a trend for an independent association with daytime 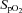 (β coefficient =−0.22, *P*= 0.059). The SS disease status, together with daytime 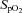 and age, explained 22% of the variance in vasoconstrictor response to IBH (*P* < 0.0001).

## Discussion

This study set out to evaluate the skin vascular responses in African children with SS disease aged between 2.7 and 12.5 years and their age-matched controls. The main finding is that blood flow and blood flow responses in the cutaneous microcirculation differ from those of healthy control children and that this is in part due to altered resting flow motion of sympathetic origin. We have further shown that children with SS disease exhibit an enhanced reflex response to an inspiratory breath hold indicative of differences in sympathetically mediated control of skin blood flow between the two study groups. Both the resting blood flux and the response to inspiratory breath hold appear to be associated with haemoglobin oxygen saturation as well as age and sickle status. Together these data support the hypothesis that children with SS disease exhibit an altered autonomic nervous system reactivity, which may be an adaptation to haemoglobin oxygen desaturation and may contribute to complications, including pain, leg ulcers and central nervous system events.

The raised skin blood flow during resting conditions in our paediatric SS disease cohort is consistent with that seen in adults with SS disease (Mohan *et al.* 1998) and supports the hypothesis that increased tissue perfusion in SS disease may be a compensatory mechanism that counteracts the effects of anaemia, suboptimal tissue oxygenation and the increased microvascular resistances due to haemorheological changes and abnormal erythrocyte adherence to the endothelium (Hebbel *et al.* 1990). Increased peripheral vasodilatation may also explain the relatively low blood pressure observed in these children, which may protect from vascular disease, at least initially (Nath *et al.* 2004); relative hypertension is ultimately associated with increased risk of stroke and death (Pegelow *et al.* 1997).

In children with SS disease, the increased resting skin blood flux that we report was associated with alterations in resting flow motion. We observed an enhanced proportion of energy in the sympathetic band (IV), which reflects enhanced autonomic reactivity (Pearson *et al.* 2005). Previous studies have shown that during increased skin blood flux there is an overall increase in total spectral power of blood flow in all spectral bands, particularly in the cardiac and respiratory components caused by vasodilatation of larger vessels (Avery *et al.* 2009; Clough *et al.* 2011; Rossi *et al.* 2011; Sheppard *et al.* 2011). In adults, the cardiogenic frequency (1 Hz) can contribute nearly 50% of the total power of the wide probe signal and thus results in a reduction of the proportion of the total signal attributable to the low-frequency intervals associated with local flow motion (Clough *et al.* 2009). Furthermore, we have demonstrated using a high-power laser Doppler flowmetry probe that the power of the cardiogenic frequency interval within the resting blood flow trace is highly dependent on the depth of sampling of the laser Doppler flowmetry probe. To limit the impact of these potential confounders we excluded the higher frequency bands from the present analysis and normalized to the total energy between bands III and VI, reflecting local flow motion. Changes in relative power spectral density of sympathetic origin have been reported in individuals with hypertension (Stefanovska *et al.* 1999; Rossi *et al.* 2011) and in diabetes (Meyer *et al.* 2003). Changes in myogenic flow motion have also been seen during local warming of the skin (Avery *et al.* 2009; Sheppard *et al.* 2011). In children with SS disease, resting skin blood flow was positively associated with the relative spectral power in the sympathetic PSD band and negatively with the myogenic frequency band. Together, these data are consistent with an enhanced perfusion of the superficial nutritive capillary bed due to a sympathetically mediated constriction of deeper arteriovenous anastamoses, which are abundantly prevalent in the finger pulp, and a reduced myogenic vasoconstrictor tone, when peripheral vessels are more dilated in SS disease. Previously, Gryglewska *et al.* ()^2010^ have shown that daytime systolic blood pressure appears to be the most consistent predictor of elevated sympathetic and myogenic flow motion. However, the children with SS disease had lower arterial blood pressure, in line with the previous literature (Pegelow *et al.* 1997), and systolic blood pressure was not a predictor in univariate analysis. The altered pattern of flow motion that we report is therefore unlikely to result from this mechanism. We saw no difference in endothelium-mediated resting flow motion in our paediatric study cohort in either of the endothelium-related PSD bands. An impaired endothelium-dependent and independent dilatation of the cutaneous circulation (Rodgers *et al.* 1984; Mohan *et al.* 1997) and an enhanced venoarteriolar reflex (Tharaux *et al.* 2002) have been reported in adults with SS disease. It is probable that a peripherally vasodilated state, together with augmentation of the local integrative control of microcirculatory blood flow, both contribute to the pathogenesis of complications associated with SS disease. The relationships found for the sympathetic frequency band might reflect a reflex increase in sympathetic activity occurring in response to a fall in ABP caused by vasodilatation driven by oxygen delivery and the level of anaemia. However, given that we saw no relationship between haemoglobin and either resting blood flux or power spectral density in the sympathetic band, we are unable to demonstrate this in our cohort.

A major new finding of the present study is that the vasoconstrictor response to inspiratory breath hold was substantially greater in children with SS than their controls. In healthy adults, the inspiratory breath hold stimuli and skin cooling have both found to be reliable manoeuvres for evoking skin vasomotor responses in routine clinical testing (Feger & Braune, 2005). The peripheral vasoconstrictor response to deep inspiration in healthy adults is mediated by an increased sympathetic activity (Allen *et al.* 2002; Rauh *et al* 2003). With autonomic failure in multisystem atrophy and pure autonomic failure there is a reduced sympathetic vasoconstrictor response to IBH compared with healthy control subjects (Young *et al.* 2006). Interestingly, our control paediatric data showed similar responses compared with previous adult control data (Khan *et al.* 1991; Asahina *et al.* 2003; Young *et al.* 2006). The enhanced magnitude of the response in children with SS disease compared with control children was also greater than responses seen in previously reported control adult populations (Khan *et al.* 1991; Asahina *et al.* 2003; Young *et al.* 2006). The increased IBH response in SS disease clearly indicated a sympathetic response and is consistent with changes in spectral power that reflect increased sympathetic drive.

The strongest predictor of vasoconstrictor response to IBH in our data was the genotype, consistent with previous data showing withdrawal of parasympathetic and relative excess of sympathetic drive in patients with SS disease (Treadwell *et al.* 2011). There is some evidence for associations with complications of SS disease, including painful crises involving the bones (Mohan *et al.* 1998) and skin ulceration (Mohan *et al.* 1997), as well as differences in the response to social stress (Treadwell *et al.* 2011). Although the control children in our study were asymptomatic, they were siblings of children with SS disease; some almost certainly had sickle cell trait, and we cannot exclude the possibility that a few had asymptomatic SS disease. Our data also suggest an effect of oxygen saturation in those with SS disease, which might be a target for therapy. Further adequately powered studies exploring the interaction with oxygen saturation specifically in patients with SS disease are warranted, and vasoconstrictor response to IBH might be an end-point in treatment trials (Kirkham *et al.* 2006).

In summary, the present study provides evidence of increased resting peripheral blood flow in children with SS disease. It contributes to the hypothesis that increased sympathetic stimulation is exhibited in SS disease and, interestingly, from a young age. We have further shown that laser Doppler fluximetry combined with vasoprovocation using an inspiratory breath hold can be used to explore autonomic reactivity in children; the protocol was easily carried out by all subjects, except one aged <3 years old, and can be used to explore predictors and associations with clinical complications, e.g. skin ulceration and vaso-occlusive crisis.

## Supporting Information

The following supporting information is available in the online version of this article (http://onlinelibrary.wiley.com/doi/10.1113/expphysiol.2011.064055/suppinfo).

**Figure S1.** Relationship between resting blood flux in theskin (RBF) and age, mean arterial blood pressure and daytime*S*_pO__2_ of 65 children with SCA (filledsquares) and 20 control children (open squares). Skin blood flowwas measured in the pulp of the index finger of the non-dominanthand using laser Doppler fluximetry. Haemoglobin oxygen saturation(*S*_pO__2_) was measured usingmotion-resistant pulse oximetry during the day at rest using a 2 saveraging time and 1 Hz sampling rate (Masimo Radical; ArtemisUK). **Figure S2.** Relationship between resting blood flux in theskin (RBF) and spectral power density (PSD) in the 0.02–0.05Hz (sympathetic) and 0.05–0.15 Hz (myogenic) frequency bandsexpressed relative to total spectral power summed across thefrequency bands 0.005 to 0.15 Hz. Measurements of blood flux weremade in 65 children with SCA in the pulp of the index finger of thenon-dominant hand using laser Doppler fluximetry. RBF waspositively associated with the sympathetic PSD band (*r* =−0.28, *P* = 0.022) and negatively with the relativespectral power in the myogenic frequency band (*r* =−0.30, *P* = 0.018) and in children with SCA.
