# Supplementary material for: Peripheral vascular response to inspiratory breath hold in paediatric homozygous sickle cell disease
Source: Exp Physiol. 2012 Jun 1;98(1):49–56. doi: 10.1113/expphysiol.2011.064055 (PMC4463767; doi:10.1113/expphysiol.2011.064055)
Supplement: Supplementary file 1 — Figure S1. Relationship between resting blood flux in theskin (RBF) and age, mean arterial blood pressure and daytimeSpO2 of 65 children with SCA (filledsquares) and 20 control children (open squares). Skin blood flowwas measured in the pulp of the index finger of the non-dominanthand using laser Doppler fluximetry. Haemoglobin oxygen saturation(SpO2) was measured usingmotion-resistant pulse oximetry during the day at rest using a 2 saveraging time and 1 Hz sampling rate (Masimo Radical; ArtemisUK). Figure S2. Relationship between resting blood flux in theskin (RBF) and spectral power density (PSD) in the 0.02–0.05Hz (sympathetic) and 0.05–0.15 Hz (myogenic) frequency bandsexpressed relative to total spectral power summed across thefrequency bands 0.005 to 0.15 Hz. Measurements of blood flux weremade in 65 children with SCA in the pulp of the index finger of thenon-dominant hand using laser Doppler fluximetry. RBF waspositively associated with the sympathetic PSD band (r =−0.28, P = 0.022) and negatively with the relativespectral power in the myogenic frequency band (r =−0.30, P = 0.018) and in children with SCA. [file eph0098-0049-sd1.doc]

**On Line Supplement**

**Peripheral vascular response to inspiratory gasp in paediatric sickle cell anaemia**

^1^Veline S L’Esperance, ^2^Sharon E Cox, ^3^David Simpson, ^1^Carolyn Gill, ^4^Julie Makani, ^4^Deogratias Soka, ^4^Josephine Mgaya, ^5,6^Fenella J Kirkham, ^1^Geraldine F Clough

**Figure S1** Relationship between resting blood flux in the skin (RBF) and age, mean arterial blood pressure and daytime SpO_2_ of 65 children with SCA (closed squares) and 20 control children (open squares). Skin blood flow was measured in the pulp of the index finger of the non-dominant hand using laser Doppler fluximetry. Haemoglobin oxygen saturation (SpO_2_) was measured using motion resistant pulse oximetry during the day at rest using a 2 second averaging time and 1Hz sampling rate (Masimo Radical – Artemis UK).

**Figure S2** Relationship between resting blood flux in the skin (RBF) and spectral power density (PSD) in the 0.02-0.05 Hz (sympathetic) and 0.05-0.15 Hz (myogenic) frequency bands expressed relative to total spectral power summed across the frequency bands 0.005 to 0.15 Hz. Measurements of blood flux were made in 65 children with SCA in the pulp of the index finger of the non-dominant hand using laser Doppler fluximetry. RBF was positively associated with the sympathetic PSD band (r=-0.28, p=.022) and negatively with the relative spectral power in the myogenic frequency band (r=-0.30, p=.018) and in children with SCA.
